# *Bifidobacterium longum* suppresses colorectal cancer through the modulation of intestinal microbes and immune function

**DOI:** 10.3389/fmicb.2024.1327464

**Published:** 2024-03-22

**Authors:** Fangjian Shang, Xia Jiang, Haobo Wang, Shang Guo, Shuo Kang, Bin Xu, Xin Wang, Shihao Chen, Ning Li, Bo Liu, Zengren Zhao

**Affiliations:** ^1^Department of General Surgery, Hebei Key Laboratory of Colorectal Cancer Precision Diagnosis and Treatment, The First Hospital of Hebei Medical University, Shijiazhuang, China; ^2^Medical Insurance Office, The Second Hospital of Hebei Medical University, Shijiazhuang, China

**Keywords:** CRC, *Bifidobacterium longum*, AOM/DSS, proliferation, invasion and migration

## Abstract

Colorectal cancer (CRC), one of the most common malignancies in the world, urgently requires more treatment strategies. Although there has been much research on probiotics, limited research has been done in treating cancer. The purpose of this study was to investigate the role of *Bifidobacterium longum* (*B. longum*) in the prevention and treatment of CRC. Through Cell Counting Kit-8 and Colony Formation Assays, 8 h and a *B. longum* count of 1 × 10^8^ CFU/ml were chosen as the best cocultivation conditions with CRC cells. The role of *B. longum* in inhibiting the progression of CRC cells was verified by a series of functional and immunofluorescence assays. For instance, *in vivo* assays have verified that *B. longum* could alleviate CRC progression. In addition, according to the results of *in vivo* assays and clinical statistical analysis, *B. longum* could reduce diarrhea symptoms. Mechanistically, by 16S and RNA sequencing, it was found that *B. longum* could affect the development of CRC by regulating the composition of gut microbes and enhancing immune function. The *B. longum* might inhibit the occurrence and development of CRC and relieve diarrhea symptoms by regulating intestinal microbes and immune function.

## Introduction

Colorectal cancer (CRC) is one of the most common malignancies in the world, with a high mortality rate and low cure rate (Bray et al., 2018). It is believed to stem from interactions between the host and the microbiota in the long term and is caused by mutations, activations, and deletions of oncogenes and tumor suppressor genes, which lead to adenoma-carcinoma (Coker et al., 2019). The majority of epithelial surfaces of our body are colonized by a vast number of microorganisms, especially the intestinal mucosa. The gut microbiota is a commensal, symbiotic and pathogenic microorganism that interacts with each other and with the host, which can affect their health (Song et al., 2020). Among many intestinal microorganisms, probiotics have undoubtedly been the focus of attention in recent years. Increasing evidence from epidemiological studies and *in vivo* models suggests that probiotic bacterial species can modulate the imbalance of gut microbiota composition, reduce the risk of cancer, and enhance the efficacy of tumor drugs (Behrouzi et al., 2022; Samanta, 2022).

*In vitro*, *in vivo* and gut microbiome studies have provided sufficient evidence of the preventive effects of probiotics for CRC. In particular, the role of *Bifidobacterium longum* (*B. longum*) has been mentioned many times. *In vitro* studies by Worthley et al. (2009) found that symbiotic supplementation comprising *B. longum* and resistant starch could induce specific beneficial changes in fecal microflora. NK and DC cells are very important in the prevention and control of autologous tumors. Fink et al. (2007) demonstrated that *B. longum* could initiate NK/DC interactions via DC maturation and the catalytic potential of NK cells to produce interferon-γ (IFN-γ), which proved the contribution of *B. longum* to tumor prevention and treatment at the cellular level (Song et al., 2018). Sivan et al. (2015) showed that *B. longum* could improve tumor control as well as anti-PD-L1 therapy. What was more noteworthy was that the combination treatment nearly abolished tumor outgrowth and increased the efficiency of the PD-L1 blocking antibody against tumors. *In vivo* studies have provided a basis for the antitumor activity of *B. longum* by using animal models (Sivan et al., 2015). Many animal models have already successfully provided insights to comprehend the link between gut microbes and CRC, such as genetic knockout, germ-free and chemical mouse models (Uttam et al., 2020). It would be interesting to employ animal models of inflammation-induced CRC for *B. longum* research to elaborate its potential benefits and to elucidate the molecular mechanism involved in their probiotics. Many articles have already used azomethane (AOM) and sodium dextran sulfate (DSS) mouse models to prove the benefit of *B. longum* (Yassin et al., 2019). It is also worth noting that in the study of probiotics, the relief of diarrhea by *B. longum* has also been a concern of many scholars, and many articles have reported the good relief of diarrhea by *B. longum* (Andresen et al., 2020).

The administration of probiotics has become a particular interest in the prevention and treatment of CRC. It should be emphasized that not all probiotics from a particular species have the same properties and will show the same effect in the organism because the probiotic strain itself is the main influencing factor (Suez et al., 2019). Fortunately, we isolated a new strain of *B. longum* from infant feces. The objective of this study was to verify the relationship between *B. longum* and the occurrence and development of CRC. Additionally, we evaluated the preliminary relationship between *B. longum* and CRC through cell function experiments and a mouse CRC model induced by AOM/DSS. Then, a small sample clinical study was conducted to explore the effect of *B. longum* on diarrhea diseases.

## Materials and methods

### Probiotic treatment

Freeze-dried living *B. longum* was kindly offered by Hebei Yiran Biotechnology Co., Ltd. for free (food production certificate No: SC13113012300078, Hebei, China). In cell assays, before *B. longum* was used in cell coculture, the culture medium was used by cell gradient dilution, rendering the probiotics at the experimental concentration. In animal experiments, PBS is used to configure the expected concentration of the probiotics.

### Cell culture

The human CRC cell lines LOVO, SW480, and SW1463 were obtained from the First Hospital of Hebei Medical University and were maintained in DMEM supplemented with 10% fetal bovine serum (FBS, Invitrogen, Grand Island, NY) and 1% penicillin (100 U/ml) with streptomycin (100 mg/ml) at 37°C in a humidified atmosphere of 5% CO2.

### Cell proliferation assay

The growth of cells was measured with Cell Counting Kit-8 (CCK-8, Boster, Wuhan, Hubei, China). The cells were plated into 96-well culture plates (2.0 × 10^3^/well) and incubated for 2 or 4 days at 37°C in a humidified incubator with 5% CO2. Every 24 h, the number of *B. longum* cells was quantified by adding 10 μl of CCK-8 to each well, followed by incubation for another 2 h. The absorbance of each well at 450 nm was measured by a microplate reader (Promega, Madison, WI, USA).

### Colony formation assay

One thousand cells were plated in culture dishes and cultured for 14 days. The culture medium was replaced on the seventh day of the experiment. The cells were fixed with 4% paraformaldehyde and stained with 0.5% crystal violet for 30 min. Then, fixed-sized colonies were selected as the standard for counting before counting the number of colonies.

### Wound-healing assay

Colorectal cancer cell lines were divided into two groups: con and *B. longum*, which were inoculated in 6-well plates at a cell quantity of 5 × 10^5^ cells per well. After 12 h, the *B. longum* group was cocultured with *B. longum* (1 × 10^8^ CFU/well) for 8 h. After that, a wound was created by a 200 μl micropipette tip before serum-free DMEM was used to wash the cells three times. The scratch width was observed and photographed at 0 h, 24 h, and 48 h after wounding by microscopy.

### Transwell assay

The migration and invasion of cells were measured by using an 8 μm Transwell chamber in 24-well plates (Corning, Waltham, MA, USA). First, a total of 650 μl of DMEM supplemented with 20% FBS was added to the lower chamber. Then, 100 μl of serum-free medium with 2 × 10^5^ tumor cells were placed into the upper chamber. The cells were incubated for 24 h at 37°C. To measure invasion, a chamber containing Matrigel (Corning, Waltham, MA, USA) was used. The remaining steps were performed according to the above method. After 24 h, the chamber was stained by diff-quick staining (BASO, Taiwan, China) and counted in five random fields. It is important to note that we selected 20% FBS because probiotics cocultured with CRC cells contain 10 FBS.

### Immunofluorescence staining

A slide was placed in each well of the 6-well plate before commencing the assay. Then, the CRC cell lines were divided into two groups: con and *B. longum*, which were inoculated in 6-well plates at a cell quantity of 5 × 10^5^ cells per well. After 12 h, the *B. longum* group was cocultured with *B. longum* (1 × 10^8^ CFU/well) for 8 h. All wells were fixed with 4% paraformaldehyde, blocked in 5% BSA, and washed with PBS for 5 min twice. The primary antibody against Ki-67 was diluted with PBS (1:250). Slides were incubated in a humidified chamber at 4°C overnight. After incubation, the slides were soaked in PBS for 2 min, which was repeated 3 times. The secondary antibodies were diluted with PBS (1:200) and were added to slides and incubated for 2 h at RT. Slides were rinsed 3 times with PBS for 2 min each. After washing, the slide was removed and placed on the fragment. Then, the cells on the slides were cultured in DAPI solution for 30 min and analyzed via an Olympus fluorescence microscope (×40).

### H&E

Mouse colorectal tissues were formalin-fixed and then paraffin-embedded, and five-micrometer sections were cut for H&E staining.

### Immunohistochemical tissue staining

Immunostaining was performed on 5-μm formalin-fixed, paraffin-embedded tissue sections using an immunoperoxidase method with rabbit anti-Ki-67 (1: 100; Sunbiote, Shanghai, China) monoclonal antibodies. Protein was visualized using PV and DAB chromogenic kits (Vector Laboratories Inc., Burlingame, CA, USA) following the manufacturer’s instructions.

### Animals

Male BALB/c mice (4 weeks old, purchased from Hebei Medical University) were maintained in large group houses under 12-h dark and light cycles and were given access to food and water. The procedures were in accordance with the guidelines for the care and use of laboratory animals from Hebei Medical University (No: 17733). After 1 week of adaptation to the environment, the animals were randomly assigned to three experimental groups. First, animals in groups 1–2 (Normal and AOM + DSS Control, *n* = 5) received only PBS; group 3 (*B. longum*, *n* = 5) received *B. longum* at 1 × 10^9^ CFU/mouse. The corresponding treatments were prepared daily and were gavaged every afternoon (0.2 mL total volume) to all mice throughout the 12 weeks. On the first day, animals in groups 2–3 were treated with a single intraperitoneal injection of azoxymethane (AOM, Sigma, St. Louis, MO, USA) 10 mg/kg, dissolved in NaCl 0.9%; then, in weeks 2, 5 and 8, 2.0% dextran sulfate sodium (DSS, Sigma, St. Louis, MO, USA) was administered *ad libitum* for 7 days. All animals were sacrificed at the 12th week after the corresponding drug was administered. Animal experiments were reviewed and approved by the Animal Committee of the First Hospital of Hebei Medical University (License number 20200326), and guidelines for the care and use of animals were followed. To analyze the inhibitory effects of the tested substances on tumor growth, tumor length (L) and width (W) were measured, and tumor volume (mm3) was calculated as [*V* = (L × W2)/2].

### 16S rRNA sequencing

16S rRNA can be used as the characteristic nucleic acid sequence of biological species, and it is considered to be the most suitable index for bacterial phylogeny and taxonomy (Caporaso et al., 2011).

### Extraction of genomic DNA

Total genomic DNA from mouse fecal samples was extracted using the ZymoBIOMICS DNA Miniprep Kit (Zymo Research, Irvine, CA, USA) according to the manufacturer’s instructions.

### Amplicon generation

Primers used for A. *lwoffii* were as follows: forward, 5′-TGGCTCAGATTGAACGCTGGCGGC-3′; reverse, 5′-TACCTTG TTACGACTTCACCCCA-3′. Primers used for *B. longum* were as follows: forward, 5′-TTCCAGTTGATCGCATGGTC-3′; reverse, 5′-GGAAGCCGTATCTCTACGA-3′. All polymerase chain reactions (PCRs) were conducted in 30-μl reactions with 15 μl of GoTaq^®^ Green Master Mix (Promega, Madison, WI, USA), with 0.2 μM forward and reverse primers and approximately 10 ng of genomic DNA. Thermal cycling for amplification of A. *lwoffii* DNA began with the initial denaturation step at 95°C for 5 min, followed by 35 cycles of denaturation at 95°C for 45 s, annealing at 67°C for 45 s, and elongation at 72°C for 60 s, and finally at 72°C for 7 min. Thermal cycling for the amplification of *B. longum* DNA began with the initial denaturation step at 94°C for 5 min, followed by 40 cycles of denaturation at 94°C for 20 s, annealing at 55°C for 20 s, and elongation at 72°C for 50 s, and finally at 94°C for 15 s.

### Agarose gel electrophoresis for PCR products

Agarose gel electrophoresis was performed on 0.7% agarose gel (SeaKem^®^ LE Agarose; Lonza, Morristown, NJ, USA) with 0.5 × Tris-acetate-EDTA as an electrophoresis buffer. Prior to cool-down of the boiled agarose, EtB“Out” Nucleic Acid Staining Solution (5 μl; YB Biotech, Taipei City, Taiwan) was added to liquid agarose (100 ml) for visualization of the separated DNA bands under ultraviolet light after electrophoresis. The DNA sample was loaded into the wells with bromophenol blue dye. The power condition was set as 130 V and 400 mA, and electrophoresis proceeded for 20 min. The DNA bands were finally photographed under ultraviolet light.

### RNA sequencing

Transcriptome sequencing is based on the Illumina sequencing platform, which plays an important role in understanding the development and disease of organisms (Wang et al., 2009). Clinical experiment. This study was conducted at the First Hospital of Hebei Medical University. The inclusion criteria of patients were as follows: patients whose IBS-SSS score was more than 175 points. The exclusion criteria included psychiatric disorders, pregnancy or breastfeeding, ingestion of probiotics or antibiotics < 2 weeks before inclusion, and unwillingness to sign the informed consent form. The primary end point was a reduction of ≥ 50 points on the IBS-SS scale. This was considered adequate to detect symptom improvement by the IBS-SS validated scoring system (Francis et al., 1997). Secondary endpoints included daily stool frequency and stool form (Bristol Stool Scale) (Plasse et al., 2020). The clinical trial was approved by the Ethics Committee of the First Hospital of Hebei Medical University (License number 20200326).

### Statistical analysis

The results of normally distributed data are expressed as the mean ± SD, while those of non-normally distributed data are expressed as the median and interquartile range. Student’s t test, one-way ANOVA and two-way ANOVA were used in this study. All statistical analyses were performed using GraphPad Prism 8 (GraphPad Software, USA) and SPSS Statistics 21 (IBM, NY, USA). *P* < 0.05 was considered statistically significant.

## Results

### Basic information of *B. longum*

*B. longum* was isolated from the intestinal tract of healthy infants. This strain has 99% similarity to *B. longum* JCM 1217 DNA, so it is classified as Lactobacillus *longum* and belongs to the food catalog strain. Supplementary Figure 1A clearly shows that the colony diameter is approximately 1–2 mm and that the edge of the colony is neat and opaque. Scanning electron microscopy (SEM) showed that the average length of *B. longum* was 1.86 μm and the width was 0.42 μm (Supplementary Figure 1B).

### The concentration and time for the coculture system of *B. longum* with CRC cells

**FIGURE 1:**
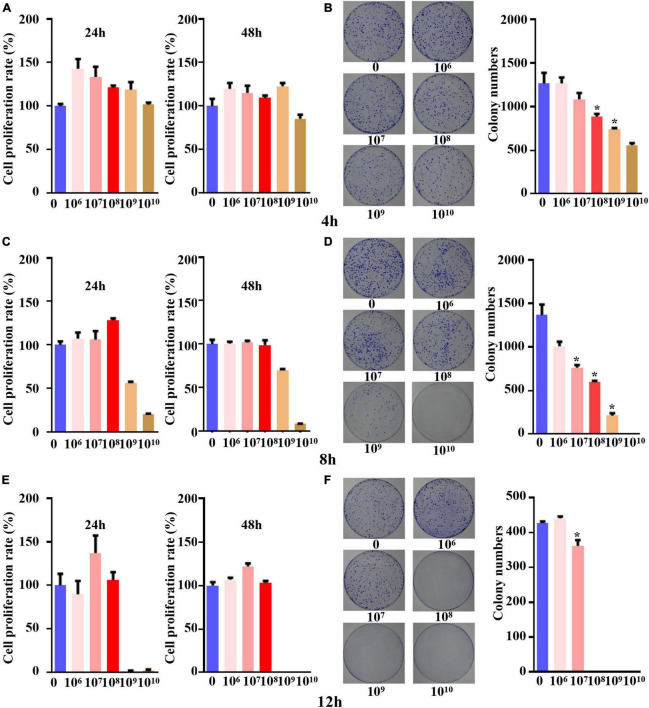
The concentration and time for co-culture system of *B. longum* with CRC cells. **(A,C,E)** Cell viability at 24 h and 48 h after co-culture for 4 h, 8 h, and 12 h was detected by CCK-8 assay. **(B,D,F)** After 4 h, 8 h and 12 h of co-culture, the colony formation at different concentrations (1 × 10^6^-1 × 10^10^ CFU/mL) was detected. *P < 0.05. Similar results were obtained from three independent experiments.

When the five different concentrations of *B. longum* were cocultured with the SW1463 cell line for 4 h, the cell proliferation was greater than the IC85 within 24 h and 48 h, indicating that the cells could grow normally with *B. longum* under this time condition (Figure 1A). The results of the colony formation assay indicated that *B. longum* had a restraining effect on the long-term survival of the SW1463 cell line compared with the control group and inhibited the proliferation and growth of colon cancer tumors when the *B. longum* count was greater than 1 × 10^8^ CFU/ml (*P* < 0.05, Figure 1B).

When the coculture time was set to 8 h, the CCK-8 assay proved that tumor cells could cogrow with *B. longum* without being affected by other factors when the *B. longum* count was between 1 × 10^6^ CFU/ml and 1 × 10^8^ CFU/ml (Figure 1C).

The results of the colony formation assay showed that when the *B. longum* count was greater than 1 × 10^7^ CFU/ml, the long-term survival of the SW1463 cell line was significantly inhibited compared with the control group (*P* < 0.05, Figure 1D).

When the coculture time was adjusted to 12 h, the CCK-8 assay showed that cells could cogrow with *B. longum* at 1 × 10^6^-1 × 10^8^ CFU/ml (Figure 1E). The results of the colony formation assay indicated that there was a statistically significant difference between the long-term survival of the CRC cell line SW1463 and the control group when the number of bacteria was greater than 1 × 10^7^ CFU/ml, which had an inhibitory effect on the growth and proliferation of tumor cells (*P* < 0.05, Figure 1F).

In summary, the short-term survival of the SW1463 cell line was not affected when the culture time was 8 h and the count of *B. longum* was 1 × 10^8^ CFU/ml, indicating that it was not caused by other factors, such as the lack of nutrients or the effects of pH value.

### Inhibitory effect of *B. longum* on the proliferation, migration and invasion of CRC cells

**FIGURE 2:**
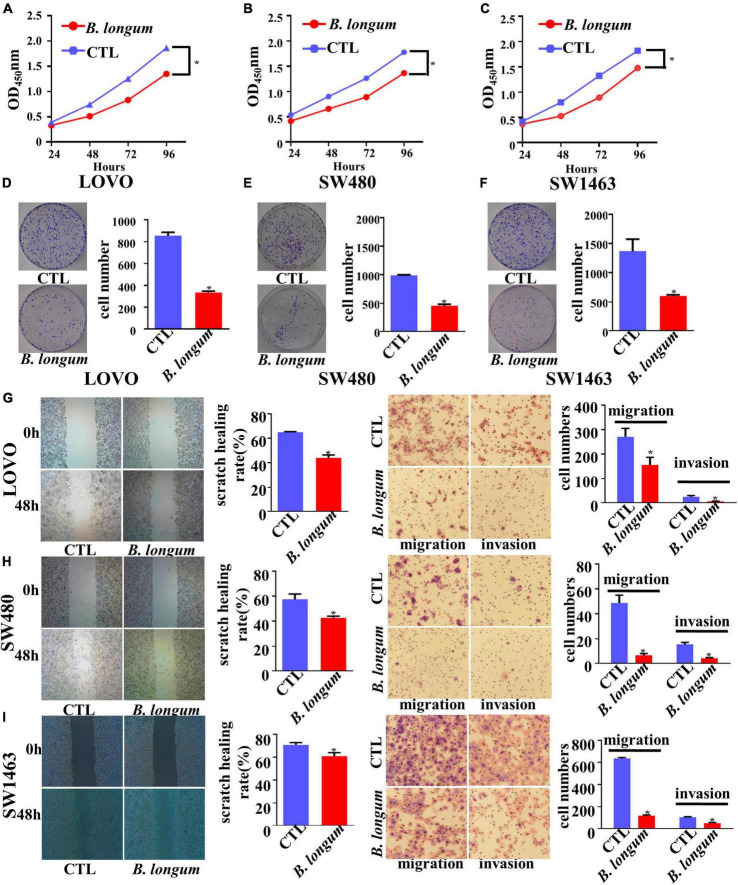
Inhibitory effect of *B. longum* on proliferation, migration, and invasion of CRC cells. **(A–F)** CCK-8 and colony formation assays comparing the effects of cell growth between the CTL and *B. longum* group in LOVO, SW480, and SW1463. **(G–I)** Wound-healing and Transwell assays comparing the effects of cell invasion and migration between the CTL and *B. longum* group in LOVO, SW480, and SW1463. *P < 0.05. Similar results were obtained from three independent experiments.

With the optimum conditions we found, we carried out a 96-h CCK-8 test and colony formation test in SW480, LOVO, and SW1463 cell lines. The results of the three groups of cell lines showed that the inhibitory proliferation of the *B. longum* group was significantly better than that of the control group (*P* < 0.05, Figures 2A–F).

Cell migration and invasion were assessed in LOVO, SW480, and SW1463 cell lines by wound-healing and Transwell assays, respectively. Through continuous scratching detection for 48 h, it was found that *B. longum* reduced the migration ability of cells. Through transwell migration and invasion results, it could be clearly seen that *B. longum* attenuated the migration of cells. In addition, *B. longum* also had the same effect on the invasive ability of cells (*P* < 0.05, Figures 2G–I).

Ki-67, a nuclear protein associated with proliferation, is often used as a mitotic index. We used immunofluorescence to observe Ki-67 expression in LOVO, SW480, and SW1463 cell lines. After the coculture of CRC cells and *B. longum*, we clearly found that Ki-67 was inhibited (Supplementary Figures 2A–C).

In summary, *B. longum* had an inhibitory effect on tumor cells at the cellular level, which also laid a good foundation for the *in vivo* assays.

### Effect of *B. longum* on the AOM/DSS CRC mouse model

**FIGURE 3:**
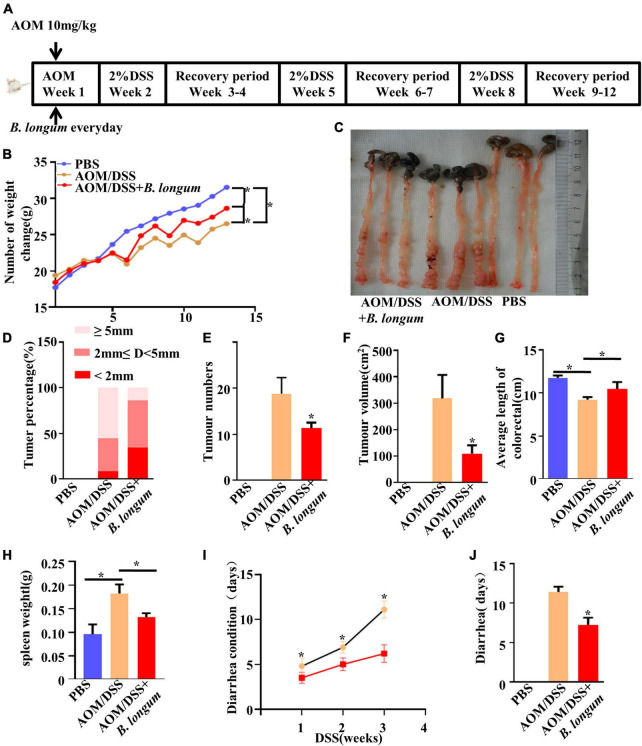
Effect of *B. longum* on AOM/DSS CRC mouse model. **(A)** Schematic representation of AOM/DSS model establishment and the Probiotics administration. **(B)** Changes in body weight of male BALB/c mice in three different treatment groups. **(C)** Tumor development, number. **(D)** Percentage of tumors in each group. **(E)** Tumor number. **(F)** Tumor volume. **(G)** Colon length in three different treatment groups. **(H)** Spleen weight. **(I)** Days of diarrhea after drinking DSS for three times. **(J)** Diarrhea days per group. *P < 0.05. Similar results were obtained from three independent experiments.

We induced colitis-associated CRC using an intraperitoneal injection of AOM followed by three cycles of DSS (Figure 3A). To evaluate the chemopreventive efficacy of *B. longum* supplementation in an AOM-DSS model, *B. longum* was added on the first day and performed once per day for the duration of this study, and body weight was determined weekly. The change in mouse body weight in the *B. longum* group was alleviated compared with that in the AOM/DSS group (*P* < 0.05, Figure 3B), which witnessed a decline in body weight after each round of DSS treatment.

The number of tumors and the length of colorectal tumors are considered to be two important evaluation indexes of the AOM/DSS mouse model (Ren et al., 2018). The length and tumor numbers in the AOM/DSS mouse model are visually reflected in Figure 3C. Compared with the AOM/DSS group, the AOM/DSS + *B. longum* group presented a significantly fewer number of tumors, tumor size and tumor volume (*P* < 0.05, Figures 3D–F).

*B. longum* significantly relieved colon shortening and splenomegaly (*P* < 0.05, Figures 3G, H). During the observation, we also found that the recovery of diarrhea caused by DSS in the *B. longum* group was faster than that in the AOM/DSS group; meanwhile, the total days of diarrhea were significantly reduced (*P* < 0.05, Figures 3I, J).

Representative photomicrographs of H&E colorectal tissue sections and Ki-67 immunohistochemistry of each group of mouse models are shown in Supplementary Figure 3. However, improved histological injury was exhibited in the *B. longum* group, which had a low level of inflammatory cell infiltration, a better mucosal architecture and shaped crypts compared with the AOM/DSS group. In addition, we evaluated the effects of *B. longum* on the expression of Ki-67 in male mouse CRC cells using immunohistochemical methods. CRC cells from the AOM/DSS group stained strongly with Ki-67, indicating a large number of highly proliferative cells. Conversely, those treated with AOM/DSS + *B. longum* showed significantly fewer Ki-67-positive cells (Supplementary Figures 3A–I).

### *B. longum* altered gut microbiota dysbiosis in AOM/DSS mice

**FIGURE 4:**
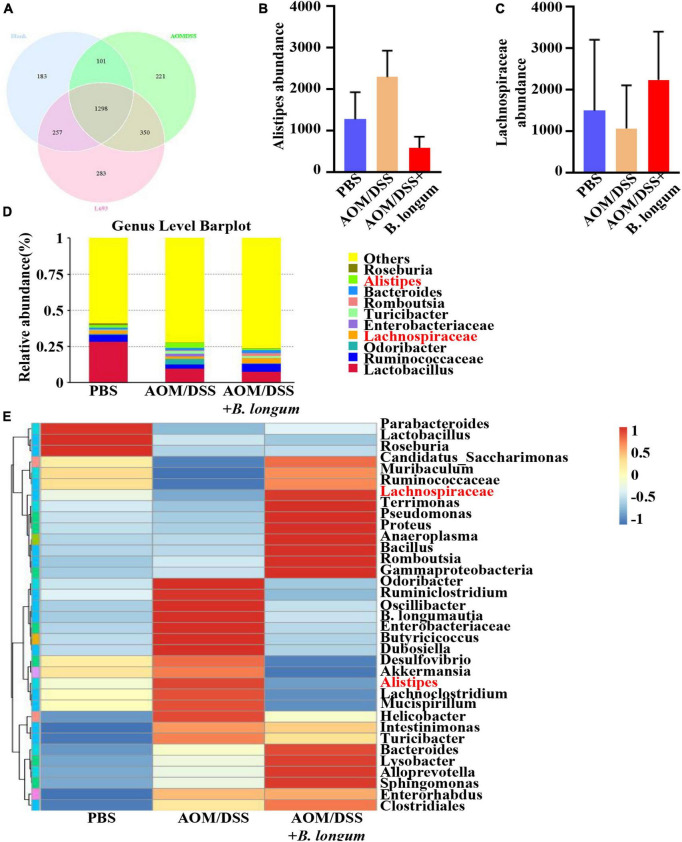
*B. longum* altered the gut microbiota dysbiosis in AOM/DSS mice. **(A)** Venn diagrams of bacterial OTUs. **(B)** The content of Alistipes in three groups. **(C)** The content of unidentified_Lachnospirace in three groups. **(D)** Bar charts at the Genus level of gut microbiota in the three groups (top 10). **(E)** The heat map of taxa in three groups. Similar results were obtained from three independent experiments.

Fecal samples were collected from the PBS, AOM/DSS and *B. longum* groups, and the fecal flora was analyzed by 16S rRNA high-throughput sequencing. The comparison of the OTUs among the three groups revealed 1839 OTUs in the PBS group, 1970 OTUs in the AOMDSS group, and 2188 OTUs in the *B. longum* group, and a total of 1298 OTUs were shared by the different samples (Figure 4A). Afterward, a key analysis was conducted at the genus level, in which it was found that there were significant differences between Alipipes and Lachnospiraceae in the three groups (Figures 4B–E).

### Mechanism of *B. longum* inhibition of AOM/DSS in an animal model

**FIGURE 5:**
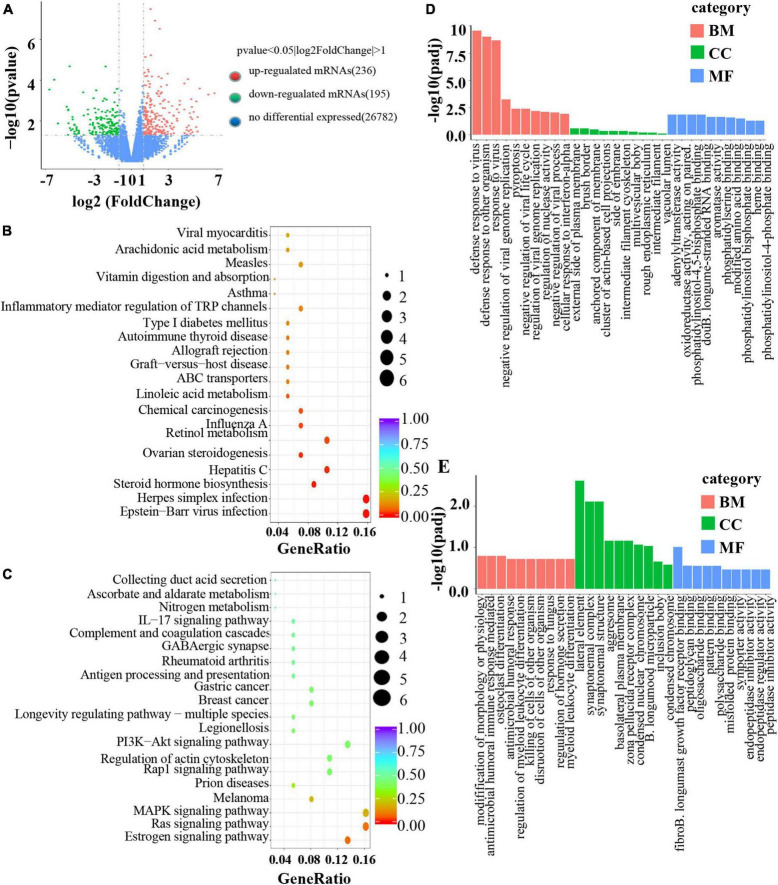
Mechanism of *B. longum* inhibiting AOM/DSS in animal model. **(A)** Volcano plot of upregulated (red spots, *n* = 236) and downregulated mRNAs (green spots, *n* = 195) from RNA sequencing data of *B. longum* vs. AOM/DSS. **(B,C)** KEGG pathway analysis based on DEGs upregulated and downregulated by *B. longum* from RNA sequencing data of *B. longum* vs. AOM/DSS. **(D,E)** GO pathway analysis based on DEGs upregulated and downregulated by BL from RNA sequencing data of *B. longum* vs. AOM/DSS.

To investigate the mechanism of *B. longum* in CRC, RNA sequencing was performed in the AOM/DSS and *B. longum* groups. A total of 431 DEGs were identified, of which 236 were upregulated and 195 were downregulated (*P* < 0.05; Figure 5A). Detailed information on the top 20 upregulated mRNAs (log2-fold change > 1; *P* < 0.05) and top 20 downregulated mRNAs (log2-fold change < −1; *P* < 0.05) is shown in Supplementary Tables 1 and 2. Through the analysis of KEGG pathways of upregulated and downregulated genes, it was found that *B. longum* affects several classical tumor pathways, as well as immune pathways and hormone pathways (Figures 5B, C). GO functional analysis of DEGs showed that the biological processes they were significantly enriched in were metabolic processes, developmental processes, signaling, growth, and biological adhesion (Figures 5D, E).

### Clinical improvement of diarrhea patients after oral administration of *B. longum*

Through the observation of diarrhea in AOM/DSS model mice, it was found that *B. longum* could relieve diarrhea symptoms very well, so we conducted a 4-week clinical comparative experiment of oral probiotics to relieve diarrhea. In this study, 20 patients with an average age of 31.05 ± 3.663 were included, including 15 males and 5 females. In the experiment, changes in the weekly IBS-SSS score, daily defecation frequency and Bristow stool score were observed (Supplementary Table 3). All patients were recorded in detail and completed the experiment.

**FIGURE 6:**
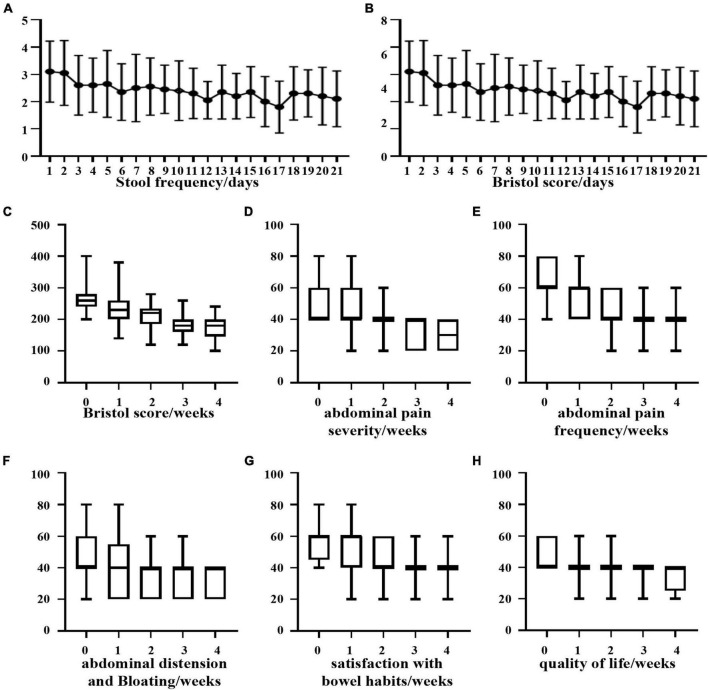
Clinical improvement of diarrhea patients after oral administration of *B. longum*. **(A)** Daily stool frequency. **(B,C)** Daily or weekly Bristol score. **(D)** Weekly abdominal pain severity. **(E)** Weekly abdominal pain frequency. **(F)** Weekly bloating frequency. **(G)** Weekly satisfaction with bowel habits. **(H)** Weekly quality of life.

Based on the statistics of the number of defecations in 21 days, it was found that although the number of defecations tended to decrease, it was not statistically significant (Figure 6A). Regarding the change in the Bristow score, we observed that there was a significant difference in the Bristow score from the 8th day compared with that from the first day, and the intake of *B. longum* improved the fecal traits (Figure 6B). Based on the individual statistics of the total IBS-SSS score and the five questions covered by IBS-SSS, we found that supplementation with *B. longum* could improve each score, and the difference between the total IBS-SSS score of the first week and that of the fourth week was more than 50 points (*P* < 0.05; Figures 6C–H).

## Discussion

With growing evidence being confirmed, it is now certain that probiotics possess numerous beneficial properties. The World Health Organization (WHO) and the Food and Drug Administration (FDA) have both reached a consensus that probiotics can be trusted as desirable dietary supplements (Ma et al., 2010). Despite previous extensive research on probiotics, their potential impact on cancer remains relatively unexplored. Therefore, it is crucial that we focus on gathering more evidence to determine the effectiveness of probiotics as a treatment and prevention method for cancer.

Ma et al. (2010) conducted a study using human colon cancer cells HT-29, DLD-1, and Caco-2 cells to investigate the potential of probiotic *Bacillus polyfermenticus* in reducing the impact of carcinogens and shrinking tumor size. Another recent report demonstrated that Streptococcus thermophilus inhibits colorectal tumorigenesis by secreting β-galactosidase. The authors employed various assays, including cell proliferation, necrosis, apoptosis, migration, invasion, and Ki-67, to support their conclusion using human colon cancer cells (Li et al., 2021). After establishing the effects of probiotics on CRC cells, the focus shifted to their impact on tumors. Tumor cells require oxygen for survival, while *B. longum* is an anaerobe. This prompted the exploration of the optimal conditions to achieve a chemical equilibrium between them. The chosen condition involved co-culturing for 8 h at a concentration of 1 × 10^8^ CFU/ml, aiming to inhibit tumor growth without inducing widespread tumor cell death. Subsequently, a series of functional assays were conducted. The results of CCK-8, colony formation, and immunofluorescence assays clearly demonstrated the inhibitory effect of *B. longum* on CRC cell growth. Similarly, the results of wound-healing and transwell assays indicated that *B. longum* also suppressed the migration and invasion of CRC cells. These findings align with the previously reported literature.

Foo et al. (2011) utilized a mouse model induced by 1,2-dimethylhydrazine to investigate the effects of *B. longum* and Lactobacillus gasser consumption. The study observed significant inhibitions of aberrant crypt foci and reductions in the number and size of tumors (Foo et al., 2011). Another study by *B. bifidum* and *Lactobacillus acidophilus* demonstrated tumor reduction through the modulation of IFN-γ and IL-10, as well as the activation of CD4 + and CD8 + cells. The researchers also assessed clinical tumor indicators such as CEA and CA199, finding that probiotics can reduce these tumor markers (Agah et al., 2019). Valadez-Bustos et al. (2019) showed that *B. longum* BAA-999 significantly reduced inflammation grade, tumor incidence, and adenocarcinoma incidence compared to the AOM + DSS group. AOM/DSS and 1,2-dimethylhydrazine are commonly used chemical models of colon cancer in mice, and the AOM/DSS model was chosen for the animal assays in this study. Our experimental results revealed that the tumor size in the *B. longum* group was smaller than that in the AOM/DSS group, which is consistent with previous experiments using the same animal models. Additionally, researchers fed mice with L. Acidophilus for 14 consecutive days and then induced murine colon adenocarcinoma CT-26 cells. After 28 days of observation and histological analyses, they found that the tumor size was nearly 50% constricted (Chen et al., 2012).

We conducted an analysis of the microbial composition and gene pathways in three different treatment groups. Our findings revealed that the microbial taxa were more abundant in the PBS and *B. longum* groups. Specifically, we observed a higher abundance of Lachnospiraceae in the *B. longum* group compared to the AOM/DSS group. Previous studies have shown that a high abundance of Lachnospiraceae is negatively associated with CRC (Flemer et al., 2018). Additionally, it has been reported that patients with colon cancer have a depletion of Lachnospiraceae compared to normal individuals (Peters et al., 2016). On the other hand, we found that the AOM/DSS group had a significantly higher abundance of Alistipes compared to the *B. longum* group. Alistipes has been identified as one of the key floras in promoting the occurrence and development of CRC (Dai et al., 2018; Parker et al., 2020). Notably, the intake of *B. longum* not only reduces cancer-promoting bacteria but also increases cancer-suppressor bacteria, demonstrating its preventive effect on CRC. Our sequencing results indicate that *B. longum* can influence various pathways, including hormone, metabolism, and immunity. Of particular interest is its impact on common tumor pathways such as the PI3K-Akt signaling pathway and MAPK signaling pathway. In future studies, we aim to further investigate the specific mechanism of action of *B. longum*.

In our animal experiments, we have observed that *B. longum* effectively alleviates diarrhea caused by DSS. This led us to investigate whether *B. longum* can improve clinical symptoms of diarrhea in patients. Our results indicate a significant positive effect. Previous studies have also reported the beneficial impact of probiotics on alleviating diarrhea symptoms. Additionally, research has shown that probiotics, particularly lactic acid bacteria and *B. longum*, have significant advantages in enhancing immunity, activating T cells, and improving the intestinal environment, thereby improving diarrhea symptoms (do Carmo et al., 2018). Chemotherapy and radiotherapy are commonly used treatments for cancer patients, often resulting in diarrhea as a side effect. Probiotics play a crucial role in alleviating these effects (Garczyk et al., 2022). Our study primarily focuses on individuals without underlying health conditions experiencing diarrhea symptoms. Although we only compared patients before and after using *B. longum*, we observed significant improvements in our main outcome measures. Our future research will delve deeper into the effects of *B. longum* on various types of diarrhea, with the hope of yielding even better results to benefit more patients.

In conclusion, *B. longum* has been found to impact the functions of CRC cells. Animal assays have shown that *B. longum* can influence the occurrence and development of CRC by regulating the composition of intestinal microbes and enhancing immune function. Furthermore, *B. longum* has demonstrated its effectiveness in regulating patients with diarrhea by restoring the balance of intestinal flora and promoting overall intestinal health. These findings highlight the significant role of *B. longum* as a probiotic in maintaining intestinal balance and promoting health.

## Data availability statement

The 16S and RNA data presented in this article have been deposited to the NCBI Repository, under accession numbers PRJNA1083191 (16S) and PRJNA1084078 (RNA).

## Ethics statement

The animal study was approved by the First Hospital of Hebei Medical University. The study was conducted in accordance with the local legislation and institutional requirements.

## Author contributions

FS: Writing—original draft. XJ: Conceptualization, Methodology, Writing—original draft. HW: Data curation, Methodology, Writing—review and editing. SG: Data curation, Methodology, Writing—review and editing. SK: Methodology, Writing—review and editing. BX: Data curation, Methodology, Writing—review and editing. XW: Data curation, Methodology, Writing—review and editing. SC: Data curation, Methodology, Writing—review and editing. NL: Data curation, Writing—review and editing. BL: Conceptualization, Funding acquisition, Supervision, Writing—review and editing. ZZ: Funding acquisition, Writing—review and editing.
