# Supplementary material for: Bifidobacterium longum suppresses colorectal cancer through the modulation of intestinal microbes and immune function
Source: Front Microbiol. 2024 Mar 22;15:1327464. doi: 10.3389/fmicb.2024.1327464 (PMC10995357; doi:10.3389/fmicb.2024.1327464)
Supplement: Supplementary file 1 [file Data_Sheet_1.docx]

The important role of *Bifidobacterium longum* in the development of colorectal cancer

Table S1. Top 20 upregulated mRNAs identified by RNA sequencing. (log_2_Fold Change>1, *P*<0.05)

| Gene_  Name | Gene_Description | Log2Fold  Change | *P* |
| --- | --- | --- | --- |
| Ddx60 | DEAD (Asp-Glu-Ala-Asp) box polypeptide 60 | 1.566049339 | 3.24E-08 |
| Ifit2 | interferon-induced protein with tetratricopeptide repeats 2 | 1.924655361 | 1.21E-07 |
| Rsad2 | radical S-adenosyl methionine domain containing 2 | 2.300204205 | 3.01E-07 |
| Ifit1 | interferon-induced protein with tetratricopeptide repeats 1 | 1.591955904 | 5.91E-06 |
| Irf7 | interferon regulatory factor 7 | 1.145886198 | 7.23E-06 |
| Ifit1bl1 | interferon induced protein with tetratricpeptide repeats 1B like 1 | 1.365996929 | 3.01E-05 |
| Ifi44 | interferon-induced protein 44 | 1.412045244 | 3.86E-05 |
| Cdhr2 | cadherin-related family member 2 | 1.071392098 | 4.20E-05 |
| Iglc2 | immunoglobulin lambda constant 2 | 2.9528334 | 4.79E-05 |
| Ighv3-5 | immunoglobulin heavy variable 3-5 | 1.289107255 | 6.12E-05 |
| Oasl2 | 2'-5' oligoadenylate synthetase-like 2 | 1.148874963 | 6.40E-05 |
| Gsdmc2 | gasdermin C2 | 1.672313254 | 6.53E-05 |
| Ighg1 | immunoglobulin heavy constant gamma 1 (G1m marker) | 2.215675339 | 6.66E-05 |
| Pla2g4c | phospholipase A2, group IVC (cytosolic, calcium-independent) | 2.451519386 | 8.04E-05 |
| Ighv1-37 | immunoglobulin heavy variable 1-37 | 4.600012473 | 8.89E-05 |
| Emp1 | epithelial membrane protein 1 | 1.382760079 | 0.000110167 |
| Btnl6 | butyrophilin-like 6 | 4.316076761 | 0.000143445 |
| Slfn4 | schlafen 4 | 1.502101109 | 0.000151074 |
| Ighv5-9-1 | immunoglobulin heavy variable 5-9-1 | 2.884529792 | 0.000170963 |
| Bglap3 | bone gamma-carboxyglutamate protein 3 | 1.045152624 | 0.000172637 |

Table S2. Top 20 downregulated mRNAs identified by RNA sequencing. (log_2_Fold Change>1, *P*<0.05)

| Gene_  Name | Gene_Description | Log2Fold  Change | *P* |
| --- | --- | --- | --- |
| Xlr3b | X-linked lymphocyte-regulated 3B | -5.009144103 | 2.10E-05 |
| Gapdh | glyceraldehyde-3-phosphate dehydrogenase | -2.092013886 | 2.14E-05 |
| Lypd3 | Ly6/Plaur domain containing 3 | -6.267315206 | 9.41E-05 |
| Sumo2 | small ubiquitin-like modifier 2 | -1.169215125 | 0.000266352 |
| Xlr3c | X-linked lymphocyte-regulated 3C | -6.652659696 | 0.000289989 |
| Gm45196 | predicted gene 45196 | -2.847786828 | 0.000313245 |
| Pak7 | p21 (RAC1) activated kinase 7 | -3.906278925 | 0.000393553 |
| Rpl30 | ribosomal protein L30 | -1.359859059 | 0.000407701 |
| Pld5 | phospholipase D family, member 5 | -3.066129928 | 0.000763483 |
| Reg3b | regenerating islet-derived 3 beta | -2.173434566 | 0.000796202 |
| Tspyl5 | testis-specific protein, Y-encoded-like 5 | -2.008722981 | 0.000898434 |
| Krt35 | keratin 35 | -2.304348353 | 0.001035498 |
| Gbx2 | gastrulation brain homeobox 2 | -1.368847186 | 0.00120736 |
| Lrg1 | leucine-rich alpha-2-glycoprotein 1 | -1.119239049 | 0.001776793 |
| Adamts18 | a disintegrin-like and metallopeptidase (reprolysin type) with thrombospondin type 1 motif, 18 | -5.249729097 | 0.001988282 |
| Fgf23 | fibroblast growth factor 23 | -2.96624829 | 0.002386434 |
| Slc16a8 | solute carrier family 16 (monocarboxylic acid transporters), member 8 | -1.517493267 | 0.002540616 |
| Stxbp5l | syntaxin binding protein 5-like | -5.121026535 | 0.002720648 |
| Ccdc65 | coiled-coil domain containing 65 | -2.673028076 | 0.002732452 |
| Gm11629 | predicted gene 11629 | -3.524534924 | 0.002737098 |

Table S3. Basic information about the patient.

| Table x | Condition of patients |
| --- | --- |
| Characteristic |  |
| Age(years) | 31.05**±**3.663 |
| Sex (%) |  |
| Female | 5(25%) |
| Male | 15(75%) |
| IBS-SSS(0 day score/ Treatment endpoint) | 271(240,280)**/**172(145,200) |
| Stool frequency (0 day /Treatment endpoint) | 3.1(2,4)**/**2.1(1,2.75) |
| Bristol stool form scale (0 day score/ Treatment endpoint) | 6(6,6)**/**4.4(4,5) |


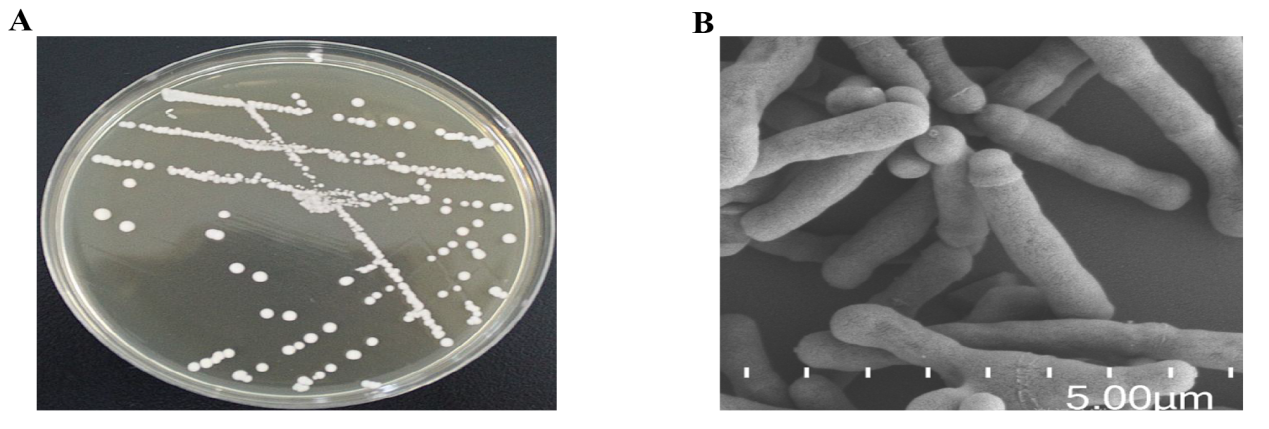


Figure S1. Basic information of *B. longum*. (A) Basic situation of *B. longum* colony. (B) SEM image of *B. longum*.


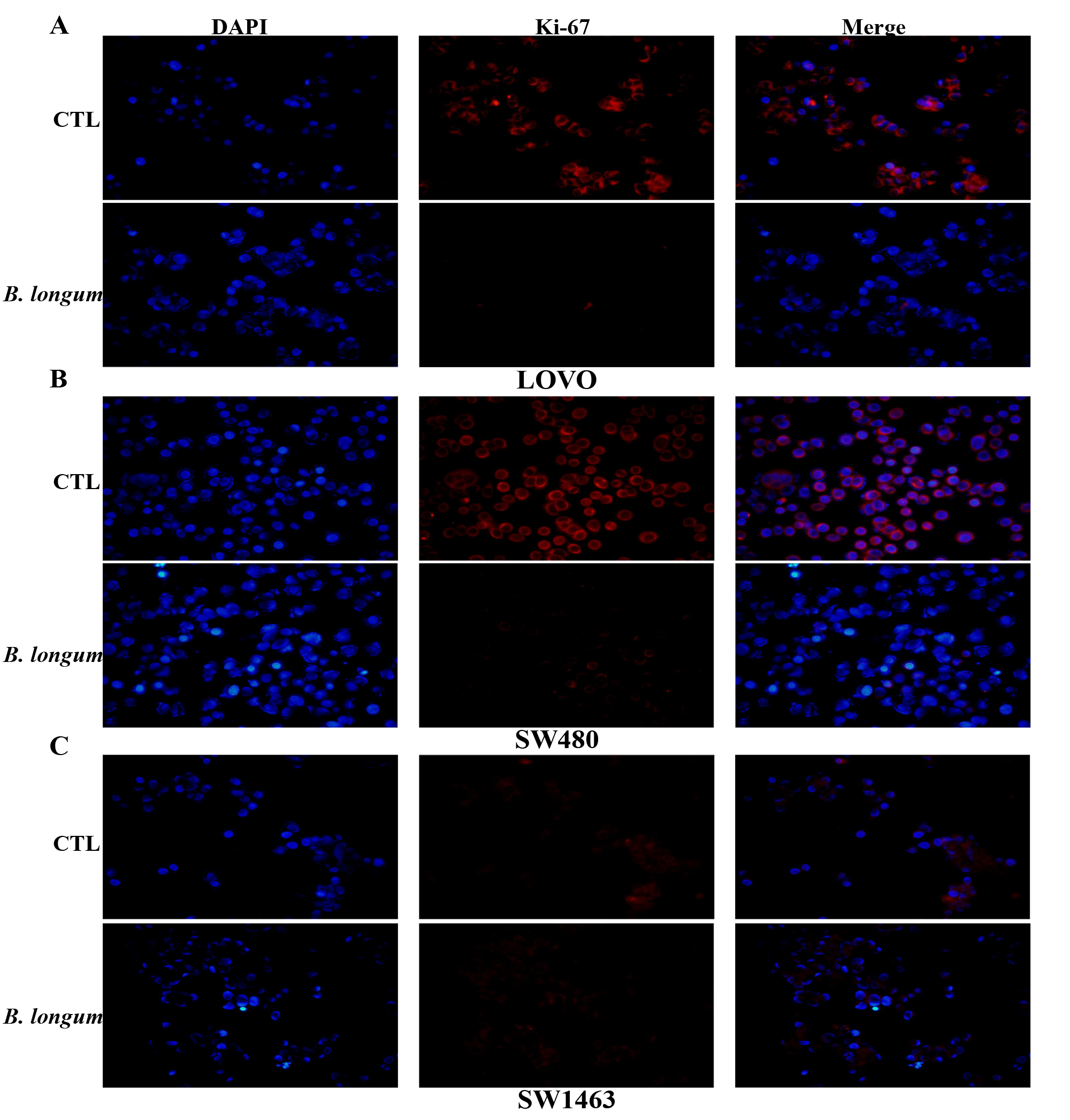


Figure S2. The expression of Ki-67 after co-culture of BL and CRC cells for 8 h was detected by immunofluorescence. (A) LOVO cell lines (× 40). (B) SW480 cell lines(× 40). (C) SW1463 cell lines(× 40).


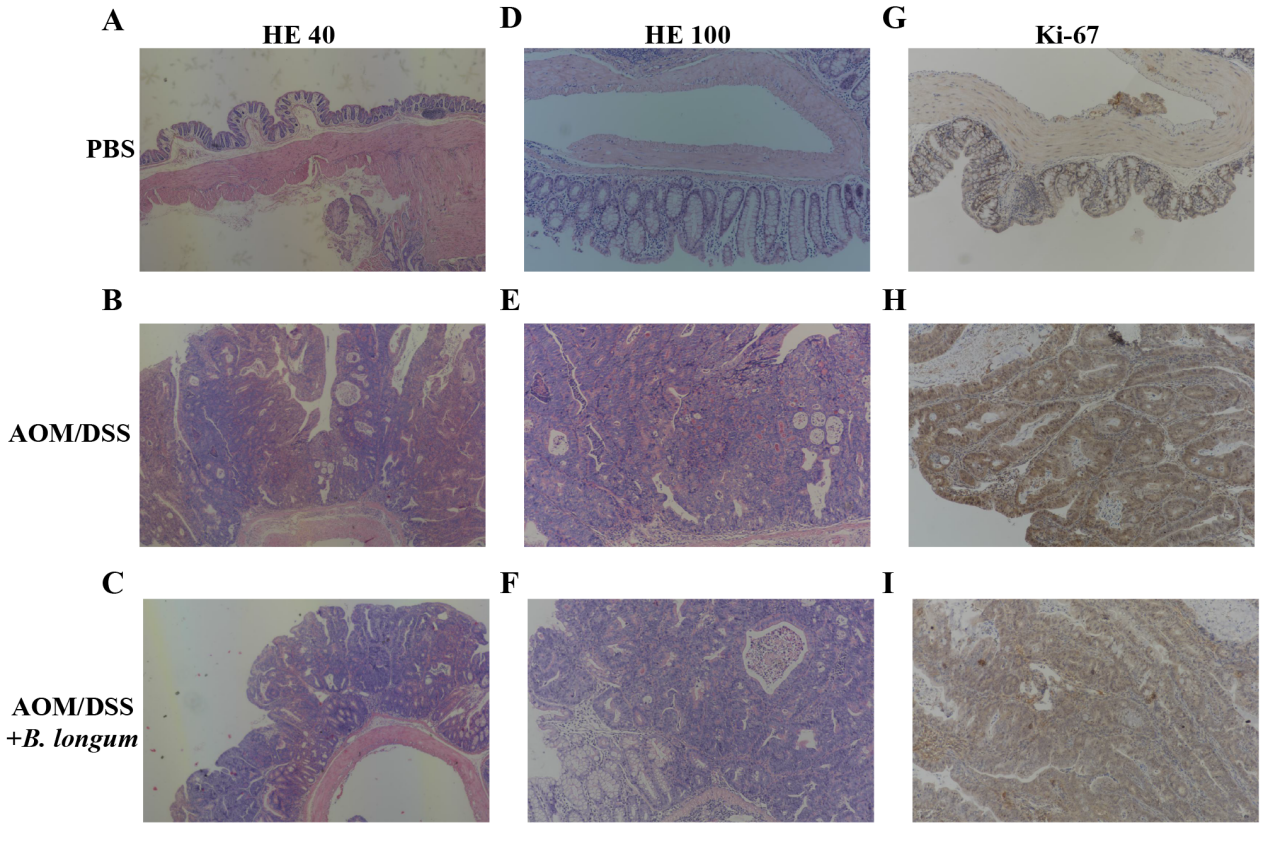


Figure S3. The HE and immunohistochemistry of Ki-67 in each group of male BALB/c mice in AOM/DSS model. (A) Normal colorectal tissue from PBS group (× 40, normal colorectal tissue). (B) Colorectal adenoma from AOM/DSS group (× 40; severely dysplastic). (C) Colorectal adenoma from AOM/DSS+*B. longum* group (× 40; moderately dysplastic). (D) Normal colorectal tissue from PBS group ( × 100, normal colorectal tissue). (E) Colorectal adenoma from AOM/DSS group (×100; severely dysplastic). (F) Colorectal adenoma from AOM/DSS+*B. longum* group (×100; moderately dysplastic). (G) Ki-67 immunohistochemistry of Normal colorectal tissue from PBS group. (H) Ki-67 immunohistochemistry of colorectal adenoma from AOM/DSS group. (I) Ki-67 immunohistochemistry of colorectal adenoma from AOM/DSS+*B. longum* group.
